# New Insights into Red Blood Cell Microcytosis upon mTOR Inhibitor Administration

**DOI:** 10.3390/ijms22136802

**Published:** 2021-06-24

**Authors:** Justyna Jakubowska, Bartłomiej Pawlik, Krystyna Wyka, Małgorzata Stolarska, Katarzyna Kotulska, Sergiusz Jóźwiak, Wojciech Młynarski, Joanna Trelińska

**Affiliations:** 1 Department of Pediatrics, Oncology and Hematology, Medical University of Lodz, ul. Sporna 36/50, 91-738 Lodz, Poland; justyna.magdalena.jakubowska@umed.lodz.pl (J.J.); bartlomiej.pawlik@umed.lodz.pl (B.P.); krystyna.wyka@umed.lodz.pl (K.W.); malgorzata.stolarska@umed.lodz.pl (M.S.); wojciech.mlynarski@umed.lodz.pl (W.M.); 2 Postgraduate School of Molecular Medicine, Medical University of Warsaw, 02-091 Warsaw, Poland; 3 Department of Neurology & Epileptology and Pediatric Rehabilitation, The Children’s Memorial Health Institute, ul. Dzieci Polskich 20, 00-999 Warsaw, Poland; K.KOTULSKA@IPCZD.PL; 4 Department of Child Neurology, Medical University of Warsaw, ul. Banacha 1A, 02-097 Warsaw, Poland; sergiusz.jozwiak@wum.edu.pl

**Keywords:** everolimus, mTOR inhibitor, microcytosis of the red blood cells, K562 cells

## Abstract

The aim of this study was to evaluate the effect of everolimus, a mammalian target of rapamycin (mTOR) inhibitor, on red blood cell parameters in the context of iron homeostasis in patients with tuberous sclerosis complex (TSC) and evaluate its effect on cell size in vitro. Everolimus has a significant impact on red blood cell parameters in patients with TSC. The most common alteration was microcytosis. The mean MCV value decreased by 9.2%, 12%, and 11.8% after 3, 6, and 12 months of everolimus treatment. The iron level declined during the first 3 months, and human soluble transferrin receptor concentration increased during 6 months of therapy. The size of K562 cells decreased when cultured in the presence of 5 μM everolimus by approximately 8%. The addition of hemin to the cell culture with 5 μM everolimus did not prevent any decrease in cell size. The stage of erythroid maturation did not affect the response to everolimus. Our results showed that the mTOR inhibitor everolimus caused red blood cell microcytosis in vivo and in vitro. This effect is not clearly related to a deficit of iron and erythroid maturation. This observation confirms that mTOR signaling plays a complex role in the control of cell size.

## 1. Introduction

An important category of immunosuppressive and anticancer drugs is that of mammalian target of rapamycin (mTOR) inhibitors, i.e., rapamycin (sirolimus) and their analogues everolimus, temisirolimus, and deforolimus. Rapalogs bind to FK506-binding protein-12 (FKBP12). This complex inhibits the mTOR regulatory protein kinase signal transduction pathway, which regulates cell growth, proliferation, and survival [1]. mTOR can form two functionally distinct complexes that differ in their sensitivity to rapalogs. mTOR complex 1 (mTORC1) regulates translation and cell growth via phosphorylation of S6 kinase and eukaryotic initiation factor eIF4E binding protein (4E-BP) and is very sensitive to inhibition by rapamycin. The second mTOR complex (mTORC2) is resistant to rapamycin and is involved in organization of the actin cytoskeleton [1]. The immunosuppressive properties of mTOR inhibitors allow them to be used in solid-organ transplantation to prevent acute rejection [2,3]. They also possess antineoplastic activities, which provide effective treatment of many cancers (renal carcinoma, advanced breast cancer, neuroendocrine tumors of pancreatic origin) [4]. There are also some reports about mTOR inhibitors as candidate drugs against viral infections such as HIV or SARS-CoV-2 [5,6]. However, until now, its most promising clinical application is in the treatment of tuberous sclerosis complex (TSC), a rare disease where a mutation of the *TSC1* or *TSC2* gene leads to overactivation of mTORC1, resulting in the growth of benign tumors in multiple organs including the brain, kidney, heart, and skin [7].

Three randomized, placebo-controlled phase III trials have demonstrated the efficacy of everolimus in the therapy of subependymal giant cell astrocytoma (SEGA), angiomyolipoma (AML), and epilepsy in patients with TSC [7,8,9]. The most common side effects reported in those trials were stomatitis, infections, and metabolic and hematologic toxicities [10]. Most of the adverse hematological effects of mTOR inhibitor use are reported by organ transplant recipients and cancer patients [11,12]; however, similar effects have also been noted in TSC patients [13,14,15]. mTOR inhibitor use is also known to lead to microcytic anemia, which has been a subject of recent interest, partly due to its ubiquity and partly due to its obscurity. Many reports suggest that microcytosis is related to iron homeostasis. Other mechanisms, such as suppression of bone marrow, diminished globin production, and resistance to erythropoietin have also been considered [11].

The aim of the present study was to evaluate the effect of everolimus treatment on red blood cells parameters in the context of iron homeostasis in patients with tuberous sclerosis complex and to evaluate the effect of everolimus on cell size in vitro.

## 2. Results

### 2.1. Everolimus Has a Significant Impact on Red Blood Cell Parameters in Patients with TSC

**Figure 1:**
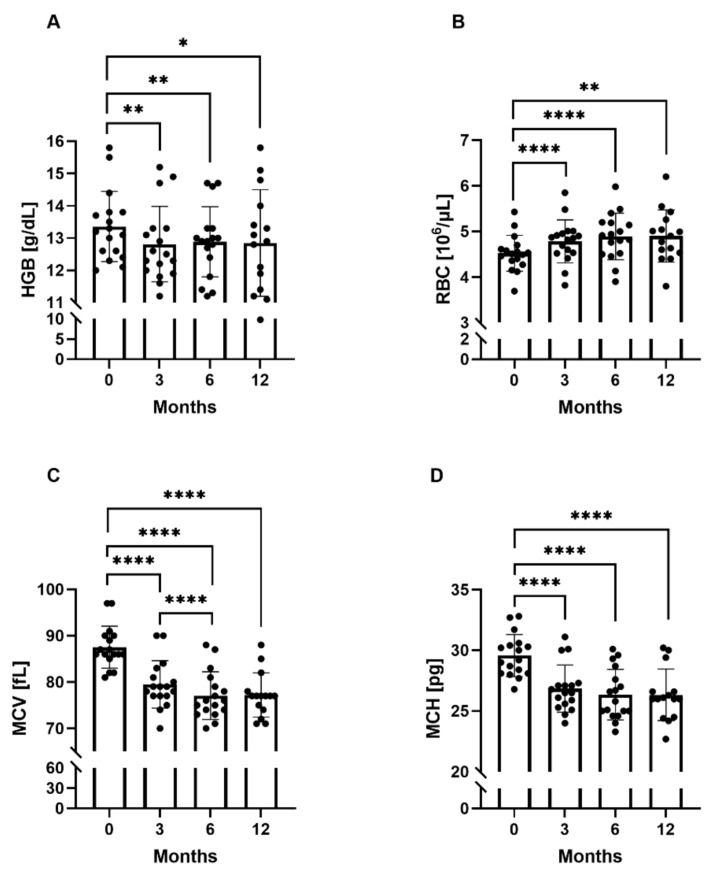
Red blood cell parameters at the study time points. (**A**) Mean hemoglobin concentrations (Hgb) at the study time points. (**B**) Mean red blood cell count (RBC) at the study time points. (**C**) Mean corpuscular volume (MCV) at the study time points. (**D**) Mean corpuscular hemoglobin (MCH) at the study time points. * *p* < 0.05, ** *p* < 0.01, **** *p* < 0.0001 (paired *t*-test).

The red blood cell parameters differed significantly between the study time points, i.e., at 3, 6, and 12 months (Figure 1). Anemia was diagnosed in one patient at 3 months (Hgb—11.9 g/dL), in three patients at 6 months (Hgb—11.2 g/dL, 11.3 g/dL, and 11.4 g/dL), and in four patients at 12 months (Hgb—9.8 g/dL, 11.1 g/dL, 11.4 g/dL, and 11.9 g/dL). Hgb, Htk, MCH decreased during the first 3 months of everolimus treatment and remained at a similar level at subsequent time points.

The MCV value was significantly lower at all three time points compared to baseline: mean (95% CL) 79.47 fL (77.00–81.00 fL), 77.06 fL (74.00–79.00 fL), and 77.20 fL (74.00–78.00 fL), for 3, 6, and 12 months, respectively, vs. 87.53 fL (86.00–90.00 fL), *p* < 10^−5^; it was also found to be significantly lower at 6 months compared to 3 months (*p* = 0.00007). The mean MCV value decreased by 9.2% after 3 months of treatment, by 12% after 6 months, and by 11.8% after 12 months. Microcytosis, defined as MCV below 80 fL, was noted in 11/17 patients (65%) at 3 months, in 13/17 patients (76%) at 6 months, and in 14/17 patients (82%) at 12 months.

RBC was significantly higher at all three time points compared to the baseline value: mean (95% CL) 4.78 × 10^9^/μL (4.53−4.95 × 10^9^/μL), *p* = 0.000064, 4.89 × 10^9^/μL (4.52−5.19 × 10^9^/μL), *p* = 0.000074, 4.90 × 10^9^/μL (4.53−5.26 × 10^9^/μL), *p* = 0.001921, for 3, 6, and 12 months, respectively, vs. 4.52 × 10^9^/μL (4.36−4.61 × 10^9^/μL). The MCHC value did not change during everolimus treatment. All statistically significant changes in red blood cell parameters at the study time points are presented in Figure 1A–D.

Baseline iron homeostasis parameters in the study group did not differ significantly compared to those in the control group (Table 1).

**Figure 2:**
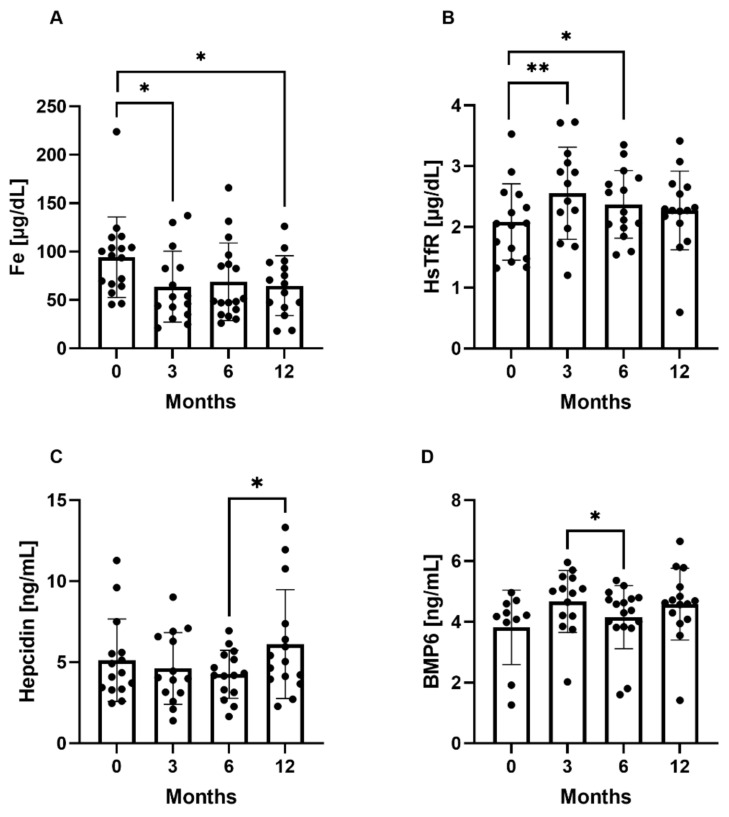
Iron homeostasis parameters at the study time points. (**A**) Mean iron (Fe) concentration at the study time points. (**B**) Mean human soluble transferrin receptor (HsTfR) at the study time points, (**C**) Mean hepcidin concentration at the study time points. (**D**) Mean bone morphogenetic protein-6 (BMP-6) concentration at the study time points. * *p* < 0.05, ** *p* < 0.01 (paired *t*-test).

In the study group, the mean iron concentration remained within the reference range during the study period. It decreased during the first 3 months of everolimus treatment from mean (95%Cl) 94.21 μg/dL (66.60–104.20 μg/dL) to 63.80 μg/dL (35.10–83.40 μg/dL), *p* = 0.01, and remained at a similar level during the subsequent time points, i.e., 68.90 μg/dL (40.30–87.00 μg/dL) and 64.72 μg/dL (42.30–88.90 μg/dL). Human soluble transferrin receptor concentration was higher at 3 and 6 months compared to the baseline value: mean (95%Cl) 2.55 μg/mL (1.98–3.06 μg/mL), *p* = 0.001016, 2.37 μg/mL (1.99–2.81 μg/mL), *p* = 0.013333, vs. 2.08 μg/mL (1.48–2.54 μg/mL). BMP-6 concentration was lower at 6 months compared to 3 months: mean (95%Cl) 4.15 ng/mL (3.84–4.76 ng/mL) vs. 4.67 ng/mL (4.19 5.44 ng/mL), *p* = 0.020693. Hepcidin concentration was higher after 12 months of everolimus treatment compared to 6 months: mean (95%Cl) 6.12 ng/mL (3.96–7.38 ng/mL) vs. 4.27 ng/mL (3.14–5.45 ng/mL), *p* = 0.021597. No statistically significant differences were noted in ferritin, IL-6, or HJV concentration between the time points (data not shown). The statistically significant differences in iron homeostasis parameters between the study time points are presented in Figure 2A–D.

### 2.2. The Decrease in Erythroid-Like K562 Cell Size In Vitro upon Everolimus Exposure Is Not the Effect of Iron Deficiency

K562 cells were treated continuously for 4 days with various concentrations of everolimus (0.005 μM to 50 μM). The status of the cells in culture was monitored every day with the trypan blue exclusion test and the colorimetric methyl-thiazol-tetrazolium (MTT) assay (Supplementary Figure S1A). Everolimus did not markedly affect the proliferation rate of K562 cells, even when used at a concentration of 25 μM. Over 70% of the cells were still able to proliferate in the presence of everolimus at concentrations ranging from 0.005 μM to 10 μM. The inhibition of K562 cell proliferation observed in response to everolimus exposure was not the effect of cell death: trypan blue exclusion analysis found that dead cells did not exceed 3% of the cells in culture, even in cultures stimulated with 10 μM everolimus for 4 days (nor shown). Moreover, K562 cells were able to recover from everolimus inhibition, as indicated by the percentage of proliferating cells markedly increasing in the presence of concentrations ranging from 0.005 μM to 10 μM, once the time of exposure was extended to 96 h.

The time- and dose-dependent changes in cell size were examined (Supplementary Figure S1C,D). The most prominent decrease in cell size was observed on day 3 upon exposure to 5 μM everolimus. Based on cell counts and cell size data, a concentration of 5 μM everolimus and 72 h of incubation time were chosen as the experimental conditions for the subsequent investigation.

**Figure 3:**
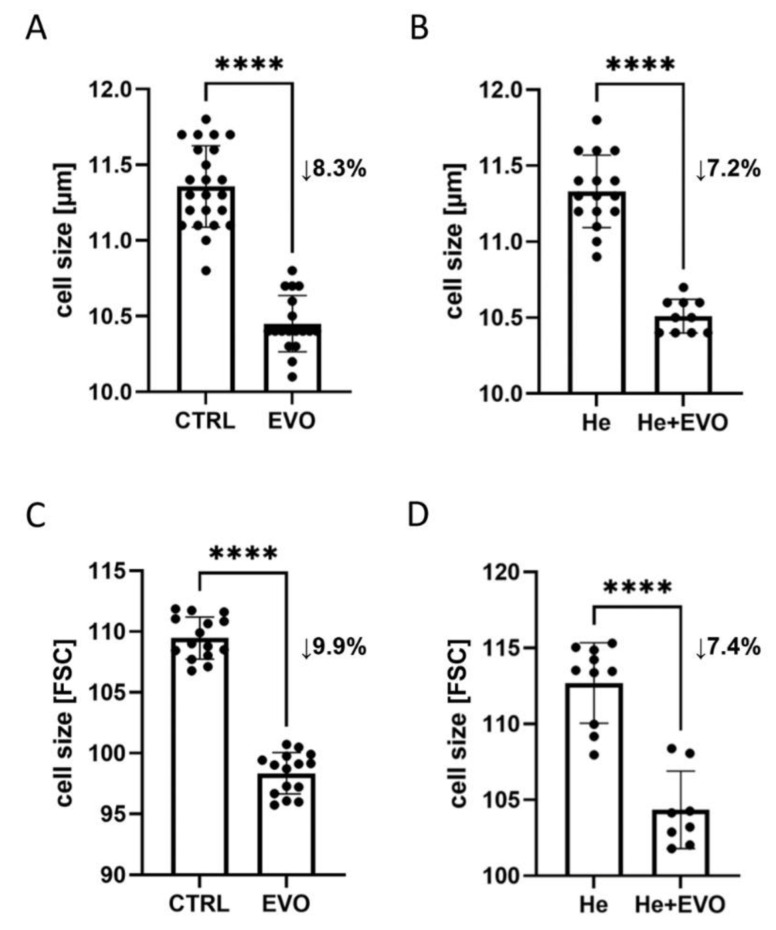
Effect of everolimus on the size of K562 cells in the presence or absence of iron. K562 cells (1 × 10^5^ cel/mL) were exposed to 5 μM everolimus alone (**A**,**C**) or in combination with 40 μm hemin (**B**,**D**) for 72 h, and differences in cell size were evaluated by the use of an automated cell counter (μm) and flow cytometry (FSC-A). CTRL—vehicle only (0.05% DMSO); EVO—5 μM everolimus; He—40 μM hemin; He + EVO—40 μM hemin + 5 μM everolimus. The results shown are the mean ± SD of three separate experiments done in triplicate. Significant differences compared to the control group are presented, **** *p* < 0.0001 (paired *t*-test for (**A**–**C**), Wilcoxon matched-pairs signed-rank test for (**D**)).

The mean cell diameter for control K562 cells, i.e., those stimulated with vehicle only (0.05% DMSO), was found to be 11.4 μm on day 3. The size of K562 cells decreased by approximately 8% during culture in the presence of 5 μM everolimus, reaching a mean diameter of 10.4 μm (Figure 3A). Similar results were obtained by flow cytometry, measuring relative differences in size between control and everolimus-stimulated cultures based on the FSC-A parameter (Figure 3C).

To check if iron administration could inhibit the tendency to a decreased cell size after everolimus exposure, the cell culture medium was supplemented with iron in the form of ferric chloride heme (hemin). Hemin alone, at a concentration of 40 μM, did not affect K562 cell size (Figure 3B,D); similarly, the addition of hemin to the cell culture with 5 μM everolimus did not prevent any decrease in cell size upon everolimus treatment. Everolimus-treated K562 cells were significantly smaller than those receiving hemin alone; this was indicated by flow cytometry measurements of median forward scatter (Figure 3C) and automated cell counter measurements of diameter in the live cell population (Figure 3B). This finding indicates that microcytosis occurring during everolimus treatment was unlikely to be related to iron deficiency since cell size was still found decreased in the presence of hemin, an iron-containing porphyrin.

### 2.3. Everolimus Affects Cells Size at Different Stages of Erythroid Maturation

To assess whether everolimus also affects the cell size of more differentiated cells, we first exposed K562 to well-known inducers of erythroid differentiation, including hemin (5, 10, 20, 50, 60 μM), cisplatin (1, 3, 5, 10, 15 μM), or doxorubicin (40, 100, 150, 200, 300 nM) for 3 days (Supplementary Figure S2). As measured by the benzidine test, hemin appeared to be the most efficient inducer of erythroid differentiation, stimulating hemoglobinization in nearly 90% of the cells. In addition, hemin did not affect cell viability and caused only minor changes in the proliferation efficiency of K562 cells. However, induction of erythroid differentiation with doxorubicin and cisplatin was accompanied by a reduction in the proliferation rate and viability of K562 cells. Since changes in proliferation rate, caused by cell cycle arrest, and cell viability, caused by necrosis or apoptosis, can influence the results of the cell size test, hemin was selected as the optimal agent for erythroid differentiation of K562 cells.

**Figure 4:**
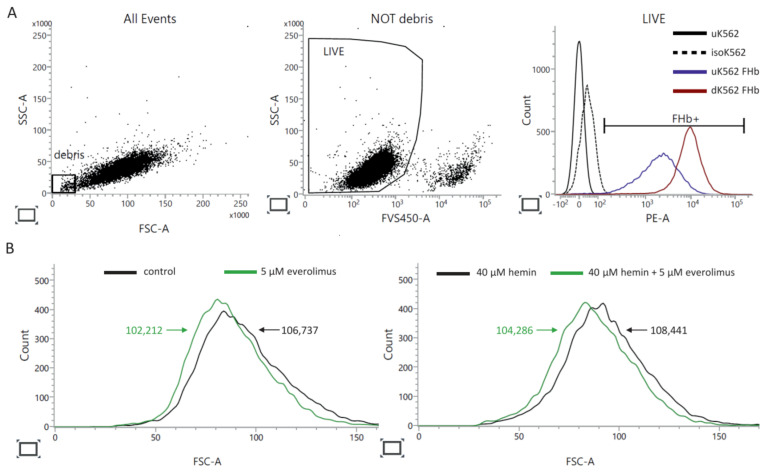
Multicolor flow cytometry analysis of fetal hemoglobin expression by K562 cells. At the end of incubation, K562 cells were harvested for analysis to determine the median FSC-A by flow cytometry. To exclude dead cells from the analysis, the cells were first stained with FVS450. After fixation (BD Cytofix/Cytoperm Buffer) and permeabilization (BD Perm/Wash Buffer), the cells were stained with PE Mouse Anti-Human Fetal Hemoglobin (FHb) antibodies (clone 2D12) or the corresponding isotype. (**A**) Presentation of gating strategy for excluding debris and dead cells from the analysis of the intracellular expression of FHb. Forward and side scatter (FSC, SSC) plot show debris at the left bottom corner of the FSC vs. the SSC density plot. The dot plot in the middle panel shows the incorporated levels of FVS450 versus SSC. Events with a low level of FVS450 incorporation represent live cells. Sample histograms (right panel) show the levels of FHb expressed by live cells. uK562—undifferentiated K562 cells, unstained (negative control), isoK562—undifferentiated K562 cells, stained with isotype (isotype control), uK562 FHb—undifferentiated K562 cells, stained for FHb, dK562 FHb—differentiated K562 cells stained for FHb. (**B**) Sample FSC-A histograms show the decrease in cell size of undifferentiated (left panel) and differentiated (right panel) K562 cells after everolimus stimulation. Flow cytometry was performed using a BD FACSLyric™ Flow Cytometer System.

After 72 h of preincubation with hemin, the differentiated cells were switched to a combination treatment with everolimus and hemin, to sustain the differentiation process, for another 72 h (Supplementary Figure S3). At the same time, control cells were subjected to the same protocol. An increase in fetal globin expression was detected in the hemin-induced cells, which confirmed the erythroid differentiation of K562 cells (Figure 4A). The size of both erythroid precursors (Figure 4B, left histogram) and more mature K562 cells (Figure 4B, right histogram) was affected by the drug, as seen by the leftward shift of the median FSC-A histograms. Everolimus-treated cells were 4.1% smaller than those of the control culture, whereas more differentiated cells were reduced in size by 4.0%. The differences in cell size values reported in Figure 3 and Figure 4 may be the results of different experimental conditions and cell processing before cytometry acquisition; this demonstrates the lability of cell size when using staining protocols including formaldehyde as a fixing agent and methanol as a permeabilizing agent.

## 3. Discussion

Our results demonstrate that the use of everolimus in patients with TSC has a significant impact on red blood cell parameters. The most common alteration was microcytosis measured by MCV; however, this did not always lead to anemia. Most fluctuations appeared to be within normal ranges, which may be the reason why this problem has not yet been explored in TSC patients during mTOR inhibitor therapy. However, significant microcytosis is a unique characteristic of mTOR inhibitor-induced anemia reported in patients after kidney and heart transplantations, as well as in cancer patients [16,17,18]. Among these patients, anemia might occur as a result of underling conditions such as cancer or kidney dysfunction. It has been proposed that the microcytic appearance of this form of anemia may be related to dysregulation in globin synthesis or failure in the autoregulatory mechanism of inflammation [11,19]. However, another widely discussed mechanism is based on disturbances in iron homeostasis [16,17].

Our in vivo approach extensively studied a range of well-established markers of iron metabolism. Our results indicate that the iron level declined during the first three months of treatment; however, the values remained within the reference range. Additionally, HsTfR concentration increased during six months of everolimus therapy. Those observations could indicate a link with iron homeostasis. However, our longitudinal analysis of other blood markers of iron metabolism did not support the hypothesis that iron deficiency is the main cause of observed microcytosis. Firstly, ferritin and IL-6 levels did not change during treatment, which excludes iron deficiency and anemia due to inflammation; in addition, most iron measurements were within the normal range. Secondly, hepcidin level did not change over six months of therapy, and the only difference was noted after 12 months. Lastly, BMP-6 and HJV proteins, connected to hepcidin production, did not correlate with hepcidin concentration. All these observations suggest that the mechanism of microcytosis due to mTOR inhibition is much more complex.

This was the basis for our further in vitro studies trying to clarify the potential mechanisms by which everolimus induces changes in the size of erythroid lineage cells. The K562 cell line, considered to be analogous to erythroid stem cells and one of the best known in vitro experimental models of erythroid cell maturation, was chosen for all experiments [20]. The size of K562 cells decreased by approximately 8% during three-day culture in the presence of everolimus. This is consistent with our in vivo observations, as the mean MCV value decreased by 9.2% after three months of everolimus treatment. Our in vivo study required a longer time to detect changes in erythrocyte size due to the fact that RBCs have a life span of 120 days and that circulating anucleated RBCs are not the target of everolimus.

mTOR, a serine-threonine kinase, serves as a nutrient availability sensor regulating cell growth and proliferation. Knight et al. reported that reticulocytes show high level of signaling through mTOR [21]. They also noted the occurrence of severe macrocytic anemia following mTORC1 activation in genetically engineered mice demonstrating over- and underactivation of mTORC1. In contrast, inhibition of mTORC1 resulted in lethal microcytic anemia. The authors conclude that the mTOR protein seems to be regulated by iron, as iron deficiency resulted in a marked reduction in mTORC1 signaling in RBCs in vitro and in vivo [21].

It has also been found that iron chelators greatly reduced mTORC1 signaling in erythroleukemic K562 cells [22]. Those observations might indicate a potential link between iron bioavailability, mTOR activity, and cell size. Culture conditions for the proliferation of K562 cells require RPMI-1640 medium supplemented with 10% (*v*/*v*) FBS (fetal bovine serum). RPMI-1640 is one of the commercial cell culture media which do not have iron in their basal formulation [23]. The only source of iron in complete RPMI-1640 medium is FBS used for its supplementation; however, even the addition of 10% (*v*/*v*) FBS to the medium could not reproduce the concentration of iron found in the plasma (10–30 µM) [24].

Therefore, in the present study, to confirm whether iron administration could inhibit the tendency to a decreased cell size after everolimus treatment, the cell culture medium was supplemented with iron in the form of ferric chloride heme (hemin). Hemin (ferriprotoporphyrin IX) is routinely employed to reduce heme deficits in porphyria patients [25]. Our findings indicate that hemin alone did not affect K562 cell size. On the other hand, the combined addition of hemin to the cell culture with everolimus did not prevent any decrease in cell size upon everolimus treatment. It is reasonable to assume, therefore, that the observed decrease in cell size of erythroid-like K562 cells in vitro upon everolimus treatment is not the effect of iron deficiency.

mTOR plays a distinct role at different stages of human erythropoiesis. In the early stage of erythroid differentiation, mTOR signaling is relatively high and promotes erythroblast proliferation. As erythropoiesis progresses, mTOR activity weakens, as reflected in the decrease in expression of mTOR downstream genes [26]. The K562 cell line is a well-known and broadly used in vitro model of erythroid maturation. Under standard culture conditions, K562 cells exhibit a low potential of hemoglobinization. However, erythroid differentiation of K562 cells can be induced by different stimuli [27,28]. We hypothesized that cells at different stages of erythroid maturation might show different requirements for mTOR signaling and hence different levels of everolimus sensitivity. In the present study, hemin was used to induce erythroid maturation of K562 cells. Hemin treatment appeared to promote erythroid differentiation, as indicated by a significant increase in the percentage of cells with a high hemoglobin level; however, no changes in erythroid differentiation of K562 cells in vitro was observed following the addition of everolimus to the cell cultures with hemin. The percentages of benzidine-positive cells did not change in cell cultures exposed to a combined treatment with hemin and everolimus in comparison to cell cultures incubated with hemin only (84.1% vs. 85.4%). Undifferentiated, and more mature, hemoglobin-producing K562 cells did not show differences in sensitivity to everolimus treatment. The everolimus treatment decreased the cell size of these two cell populations at distinct stages of erythroid maturation by the same value (4%). Our finding suggests that the stage of erythroid maturation did not affect the response to everolimus.

Although reduced mTORC1 activity is known to induce a decrease in size in many cell types, differential sensitivity to mTOR inhibitors is observed [29,30]. For example, Trendowski et al. noted that hHSCs are more sensitive to rapamycin than U937 human monocytic leukemia cells: under the same experimental conditions, a 48 h treatment with 25 nM rapamycin inhibited hHSC cell size by 24% and U937 cell size by only 12%. Rapamycin was found to have similar effects on U2OS and 293E human embryonic kidney cells (10% decrease in size) [29]. However, HeLa cells (human cervical carcinoma) appeared to be more resistant, with a decrease in size of only 4%. In the present study, K562 cells decreased in size by approximately 10% and were still able to proliferate even in the presence of 10 μM everolimus. The K562 cell line is genetically characterized by the presence of an aberrant, oncogenic Bcr–Abl kinase, which may explain the resistance of K562 cells to high doses of everolimus. The reason for this differential response to mTOR inhibitors is still unclear, but these previous findings might suggest that different cell types depend on mTOR signaling to varying degrees in the control of cell growth. Alternatively, in the case of mTOR inhibition, compensatory pathways might by activated.

## 4. Methods

### 4.1. Patients

Seventeen patients, aged 3–17 years (mean: 11.49 ± 4.69 years), with a clinical diagnosis of TSC were enrolled into the study. Patients were recruited at the Department of Pediatrics, Oncology and Hematology, Medical University of Lodz, Poland between June 2012 and August 2015. A diagnosis was made according to the TSC Consensus Conference Diagnostic Criteria [31]. The inclusion criteria comprised a clinical diagnosis of TSC, and SEGA or AML as indication for everolimus therapy. The study was conducted in accordance with the Declaration of Helsinki, and the study protocol was approved by the Bioethics Committee of the Medical University of Lodz (# RNN/306/13/KE). All individuals, or their legal representatives, gave their written informed consent to take part.

### 4.2. Treatment Protocol

Everolimus was administered orally once daily at the same time every day, consistently either with or without food. The starting dose of everolimus for children was calculated according to the body surface area (BSA): 2.5 mg for patients with BSA ≤ 1.2 m^2^; 5 mg for patients with BSA between 1.3 and 2.1 m^2^; and 7.5 mg for patients with BSA ≥ 2.2 m^2^. Everolimus whole blood trough concentration was assessed approximately 2 weeks after commencing the treatment, using ultraperformance liquid chromatography/tandem mass spectrometry (UPLC/MS/MS) [32]. Dosing was titrated to attain trough concentrations of 5 to 15 ng/mL. If the concentration was <5 ng/mL, the daily dose was increased by 2.5 mg every 2 weeks, depending on subject tolerability. The dose was reduced if trough concentrations >15 ng/mL were observed.

### 4.3. Laboratory Parameters Measurements

At the study time points (before everolimus commencement, 3 months on everolimus treatment, 6 months on everolimus, and 12 months on everolimus), the following parameters were recorded: everolimus whole blood trough concentration, hemoglobin concentration (Hgb), red blood cell count (RBC), hematocrit (Htk), mean corpuscular volume (MCV), mean corpuscular hemoglobin (MCH), mean corpuscular hemoglobin concentration (MCHC); iron homeostasis parameters: concentration of iron (Fe), ferritin, human soluble transferrin receptor (HsTfR), hepcidin, interleukin-6 (IL-6) hemojuvelin (HJV), bone morphogenetic protein-6 (BMP-6). The concentrations of iron (colorimetric method, set no 7K59-25, Abbott, Chicago, IL, USA) and ferritin (immunochemical/CLA method, test no 6K95-30, Abbott, Chicago, IL, USA) were determined by standard laboratory methods in the central laboratory of the hospital. The other parameters were determined using the ELISA method: human soluble transferrin receptor—hsTfR (EIA-4256, DRG International, Springfield, NJ, USA), hepcidin-Hepcidin-25 or H (EIA-5782, DRG International, Springfield, NJ, USA), interleukin-6—IL-6 (R&D, kit no D6050, R&D, Minneapolis, MN, USA), hemojuvelin—HJV (E-EL-HH0588, ElabScience, Wuhan, China), bone morphogenetic protein-6—BMP-6 (E-EL-HH1854, ElabScience, Wuhan, China).

The control group consisted of 47 healthy children without anemia, all of whom were sex- and age-matched with the study group. Iron homeostasis parameters were assessed in the control group and compared with those of the study group.

### 4.4. In Vitro Studies

#### 4.4.1. Reagents

RPMI 1640 medium was purchased from Lonza (Basel, Switzerland). Media supplements, penicillin, streptomycin were obtained from Sigma (MilliporeSigma, St. Louis, MO, USA). Everolimus, 10 mM solution in DMSO, was purchased from Selleck Chemicals (Houston, TX, USA). Bovine hemin powder (purity ≥ 90%) was purchased from Sigma (MilliporeSigma, St. Louis, MO, USA), and further dissolved in a 1.4 M NaOH aqueous solution to obtain 7.7 mM hemin stock solutions. Doxorubicin and cisplatin were made available for research courtesy of EBEWE Pharma (EBEWE Pharma GmbH Nfg. KG, Unterach am Attersee, Austria). Phycoerythrin-conjugated mouse anti-human fetal hemoglobin (clon 2D12), mouse IgG1, kappa isotype control, BD Cytofix/Cytoperm™ Fixation/Permeablization Kit were obtained from Becton Dickinson. PBS buffer were purchased from Corning (New York, NY, USA).

#### 4.4.2. Cell Lines and Culture Conditions

The human erythroleukemia K562 cells were purchased from the American Type Culture Collection (ATTC) (Manassas, VA, USA) and cultured at 37 °C in a humidified atmosphere of 5% CO_2_ in air in RPMI 1640 medium (LONZA, Basel, Switzerland) supplemented with 10% (*v*/*v*) heat-inactivated fetal bovine serum (FBS; Gibco, Thermo Fisher Scientific Waltham, MA, USA), 100 U/mL penicillin (Millipore Sigma, St. Louis, MO, USA), and 100 μg/mL streptomycin (Millipore Sigma, St. Louis, MO, USA). Cell proliferation and viability were determined with the trypan blue exclusion test and an automated cell counter (Countess 1 Cell Counter, Thermo Fisher Scientific, Waltham, MA, USA) each day of culture. All experiments were repeated at least three times. For each drug concentration, triplicate cultures were used. Vehicle controls of 7.3 mM NaOH for hemin and 0.05% DMSO for everolimus were run in each experiment.

#### 4.4.3. MTT Assay

K562 cells were seeded in 96-well plates at a concentration of 1 × 10^5^ cells/well in 100 μL culture medium containing escalating doses of everolimus (0.005–50 μM), hemin (5, 10, 20, 50, 60 μM), and incubated for 24–96 h at 37 °C and 5% CO_2_. For the last four hours of the experiment, 10 μL of the MTT reagent (final concentration 0.5 mg/mL) was added to each well. After the incubation period, 150 μL of acidified isopropyl alcohol (0.04 N HCl) was added into each well to solubilize the purple formazan crystals. The absorbance of the samples was read spectrophotometrically at 570 nm using a microplate reader (reference wavelength = 620 nm). Wells without cells served as negative controls, and their absorbance was subtracted from the other results. Untreated cells were the positive control. The data from the MTT assay are reported as a percentage of the control counts. Each experiment was conducted in triplicate and repeated at least three times.

#### 4.4.4. Identification of Erythroid Differentiation

##### Determination of Hemoglobin

The erythroid differentiation of K562 cells was assessed by the benzidine test. Cells (1 × 10^5^) were collected on day 3 and washed with ice-cold phosphate-buffered saline (PBS). The cells were then resuspended in 100 μL of 0.9% NaCl, and 50 μL of a solution containing 0.2% benzidine in 0.5 M acetic acid, 0.6% H_2_O_2_ was added to start the reaction. After 30 min of incubation in the dark at room temperature, 400 cells were counted by light microscopy to determine the percentage of blue heme-containing cells. Each experiment was conducted in triplicate and repeated at least three times.

##### Flow Cytometry

Fetal hemoglobin expression was monitored by flow cytometry as an erythroid differentiation marker. Cultured K562 cells (1 × 10^6^) were washed once with PBS (37 °C) to remove the excess of FBS. The cell pellets were resuspended in 1 mL of PBS, and 1 μL of BD Horizon™ Fixable Viability Stain 450 (FVS450) was added to the cell suspension for 7 min. After the washing step, the cells were fixed with BD Cytofix/Cytoperm buffer for 20 min at 4 °C. After fixation, the cells were washed twice with 1x BD Perm/Wash buffer and stained with saturating amounts of phycoerythrin-labeled fetal hemoglobin (FHb) antibody or isotype control in a final volume of 100 μL Perm/Wash buffer for 30 min in the dark at 4 °C. After the washing step, the samples were analyzed using BD FACSuite software on a BD FACSLyric flow cytometer (Becton Dickinson, Franklin Lakes, NJ, USA). The dead cells were eliminated by FVS450 staining. The level of fetal hemoglobin intracellular expression (MFI) served as an indicator of undifferentiated and more matured erythroid cells.

##### Effects of Everolimus on the Size of Undifferentiated K562 Cells

For the experiments, K562 cells were seeded at 5 × 10^4^/mL; after 24 h, everolimus or hemin was added, alone or in combination. Changes in cell size were assessed in parallel with two methods, each day during four-day culture. Since cell preparation may affect cell size, the cells were tested immediately after being removed from the culture without any further cell processing.

##### Effects of Everolimus on More Mature K562 Cells

K562 cells were first stimulated to differentiate with 40 μM hemin for 3 days. After that time, cells were transferred to a 6-well plate. Since the hemin-induced differentiation is reversible, the drug at 40 μM was added the same day to uphold the differentiated state of K562 cells. Simultaneously, the control cells, incubated with vehicle only, were processed. After the next 48 h, 5 μM everolimus was added to part of the wells of the plate with control and differentiated cells. After 3 days of incubation in the presence of everolimus, changes in cell size were evaluated.

##### Cell Size Testing

Changes in cell size were assessed by the measurement of the average cell diameter in microns with an automated cell counter (Countess 1 Cell Counter, Thermo Fisher Scientific, Waltham, MA, USA), and the relative differences in size based on the FSC-A parameter (forward scatter) by flow cytometry (FACSLyric, Becton Dickinson, Franklin Lakes, NJ, USA). Both methods enable the exclusion of debris and dead cells from the cell counts, eliminating the risk of their impact on the results. Dead cells (taking up trypan blue) were excluded from the analysis with an automated cell counter. In flow cytometry, dead cells were excluded based on FSC/SSC scattergram or high level FVS450 incorporation.

### 4.5. Statistical Analysis

Statistical comparisons between two groups were made using GraphPad Prism 9.0 software (GraphPad Software, Inc., La Jolla, CA, USA). Student’s t test was used if the data were normally distributed, as determined by the Shapiro–Wilk test. A probability value of < 0.05 was considered to be significant.

## 5. Conclusions

Our results have confirmed that everolimus causes red blood cells microcytosis (in vivo and in vitro). We have proved that microcytosis is not the effect of iron deficiency. We conclude that microcytosis upon everolimus treatment appears regardless of the stage of erythroid maturation. This observation confirms that mTOR signaling has a complex influence on cell size.

## Figures and Tables

**Table 1:** Comparison of age and iron homeostasis parameters between study (*n* = 17) and control group (*n* = 47).

Mean Value	Study Group	Control Group	*p* Value
age	11.49 ± 4.69	8.77 ± 5.04	0.06
Fe (μg/dL)	94.21 ± 41.60	82.16 ± 50.76	0.41
Ferritin (μg/L)	37.80 ± 27.15	35.24 ± 23.91	0.74
HsTfR (μg/mL)	2.08 ± 0.63	1.76 ± 0.72	0.15
IL-6 (pg/mL)	3.22 ± 5.14	1.80 ± 0.71	0.24
Hepcidin (ng/mL)	5.13 ± 2.54	5.00 ± 3.62	0.90
HJV (ng/mL)	1.23 ± 0.83	1.50 ± 1.06	0.49
BMP-6 (ng/mL)	3.82 ± 1.22	4.18 ± 1.59	0.54

Fe—iron, HsTfR—human soluble transferrin receptor, IL-6—interleukin-6, HJV—hemojuvelin, BMP-6—bone morphogenetic protein-6.

## Supplementary Materials

The following are available online at https://www.mdpi.com/article/10.3390/ijms22136802/s1.
