# Supplementary figures and images for: New Insights into Red Blood Cell Microcytosis upon mTOR Inhibitor Administration
Source: Int J Mol Sci. 2021 Jun 24;22(13):6802. doi: 10.3390/ijms22136802 (PMC8268656; doi:10.3390/ijms22136802)

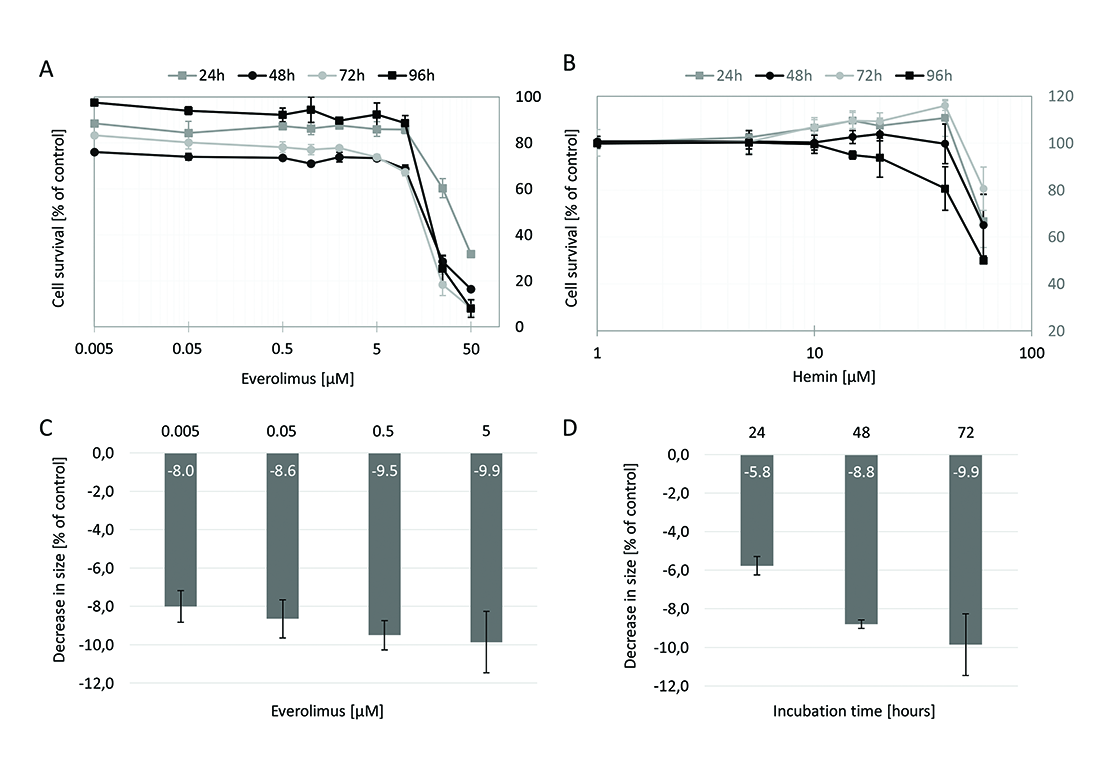

Supplement: Supplementary file 1 [file ijms-22-06802-s001.zip › Supplementary Figure 1.tif]

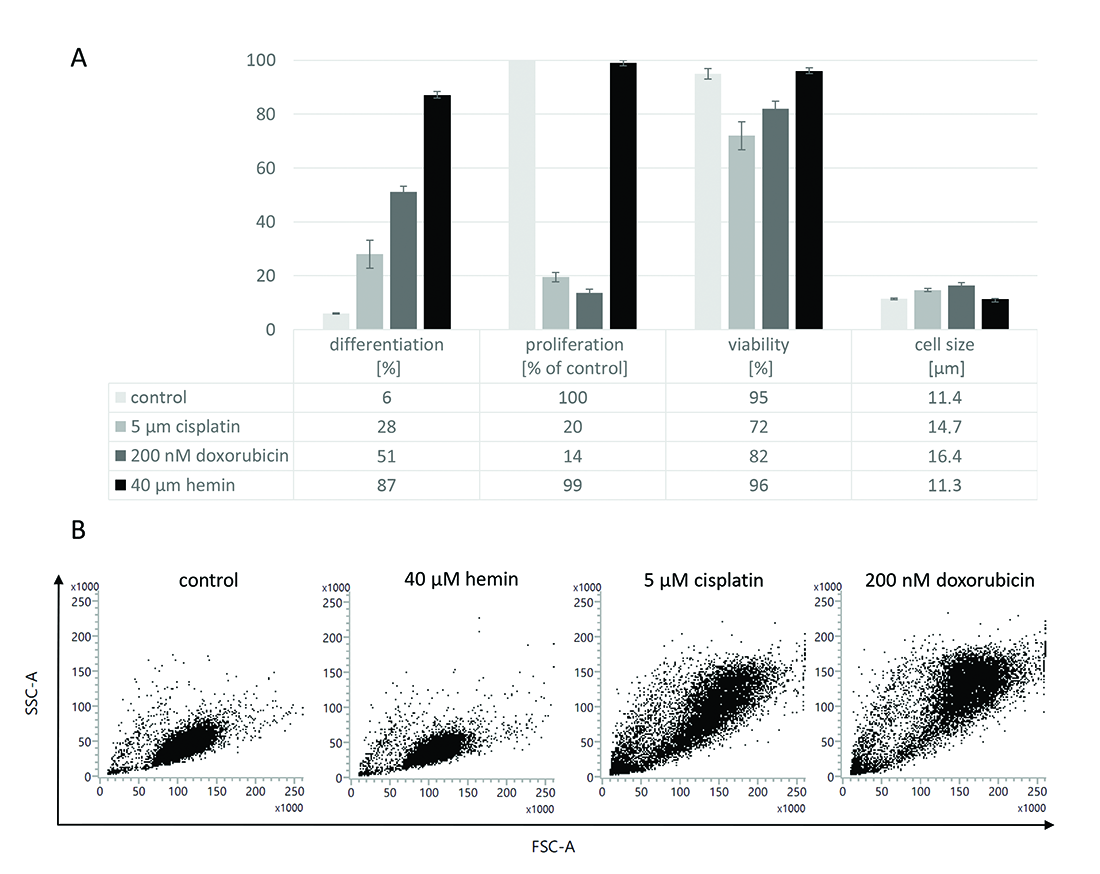

Supplement: Supplementary file 1 [file ijms-22-06802-s001.zip › Supplementary Figure 2.tif]
